# A Value-Based Steering Model for Healthcare

**DOI:** 10.3389/frhs.2021.709271

**Published:** 2021-11-26

**Authors:** Laura J. Pitkänen, Riikka-Leena Leskelä, Helena Tolkki, Paulus Torkki

**Affiliations:** ^1^Department of Public Health, Faculty of Medicine, University of Helsinki, Helsinki, Finland; ^2^Nordic Healthcare Group, Helsinki, Finland; ^3^Faculty of Management and Business, Tampere University, Tampere, Finland

**Keywords:** outcomes, value-based healthcare, effectiveness, steering, commissioning

## Abstract

This article aims to answer how a commissioning body can steer health services based on value in an environment where the commissioner is responsible for the health services of a population with varying health service needs. In this design science study, we constructed a value-based steering model consisting of three parts: (1) the principles of steering; (2) the steering process; and (3) Value Steering Canvas, a concrete tool for steering. The study is based on Finland, a tax-funded healthcare system, where healthcare is a public service. The results can be applied in any system where there is a commissioner and a service provider, whether they are two separate organizations or not. We conclude that steering can be done based on value. The commissioning body can start using value-based steering without changes in legislation or in the present service system. Further research is needed to test the model in practice.

## Introduction

During this century, outcomes have become more of a hot topic in healthcare, especially since Porter and Teisberg (1) introduced the concept of value, meaning patient-relevant outcomes in relation to the costs of delivering these outcomes. In health economics, a similar concept is known as cost-effectiveness (2). What Porter adds to the discourse is an emphasis on relevance of the outcomes to the patient (3) as opposed to general outcomes such as 5-year survival or percentage of reoperations. The rising trend of patient-centeredness (4) also contributes to this discourse. What this means from the point of view of outcomes measurement is essentially that outcomes must be measured on the patient level, not on the population level or producer level, and that outcomes measurement should include patient-relevant measures, which often means Patient-Reported Outcome Measures (PROMs). Porter et al. (5) suggests that value should be measured by the patient's medical condition, that is, the measurement of value should be tailored for each diagnosis. ICHOM (International Consortium for Health Outcomes Measurement) aims to standardize health outcomes measurement by creating diagnosis-specific standard sets of measures, which usually include both PROMs and clinical measures, sometimes also clinician-reported outcome measures (6).

There are many potential use cases for the outcomes data. Patients may use provider-level outcomes data to choose the best provider for them (7). Healthcare professionals may use the data of individual patients to monitor their development and to guide their care (8). Managers of healthcare professionals may use the data to benchmark and assess the performance of each professional to motivate them to actively work for better patient outcomes, and other such management-related uses (9). Healthcare service provider organizations may use the data to improve their performance (9, 10). And finally, the commissioner of the services may use the data to steer the service providers toward better health outcomes—which is in the center of this study.

However, there is valid criticism aimed at Porter's value-based healthcare (VBHC). Groenewoud et al. (11) note that VBHC emphasizes competition, the suitability of which in a publicly-funded healthcare system is debatable. Mjåset et al. (12) studied VBHC in four different healthcare systems and found that different elements of the concept work in different settings. Thus, it seems necessary to adapt the concept to suit the local context. Secondly, as VBHC is disease-specific and originates from specialized health care, it provides only a part of the solution for an outcomes-based healthcare system. Torkki et al. (13) argue that when the population is segmented based on their service needs, VBHC (as implemented through ICHOM standard sets) is directly applicable to only a portion of the population: the patients with a curable ailment, where the treatment is process-like and finite; and those with a single chronic condition. The rest are either multimorbid, making it difficult to base outcomes measurement on the medical condition, or their health needs are too minor (or merely preventive) to be measured through most PRO measures. Thus, VBHC in its present form is not suitable for measuring the outcomes for a large share of the population. Furthermore, the term “value” itself can be misleading, as it has many different definitions across disciplines. In this article, we rely on Porter's definition (1): value is cost-effectiveness, with an emphasis on patient-relevant outcomes.

Healthcare systems vary in terms of their financing structure. They are usually classified into three categories: the Beveridge model (national health service), the Bismark model (private insurance), and national health insurance (14). In the Beveridge model, services are financed through taxes and provided by government-owned and/or private bodies. In the insurance models, the financing is either through a government-run insurance program that every citizen pays into, or through private insurance companies where participation is mandatory. In the insurance models, provision of services is usually done by private hospitals and general practitioners contracted by the insurance companies. In reality, the system is often a combination of these elements. Regardless of the financing structure of the healthcare system, there is a payer (or multiple payers): a commissioner of the services, responsible for the service provision for a certain population—be it a regional population base or a group of insurance holders. Thus, it is in the interests of the commissioner to maximize the cost-effectiveness of the services. Also, in every system there is a commissioner paying the services and a provider producing the services, even if they are organizationally the same. Therefore, the commissioner can affect the patients' outcomes only through the service providers. In order to do so, the commissioner must use policy instruments to steer the providers.

The Finnish healthcare system is mainly tax-based with multiple payers: municipalities are responsible for primary healthcare and social care, while larger federations of municipalities are responsible for secondary and tertiary healthcare. Finland is about to undertake a major reform on its healthcare system, after which 20 welfare areas will be responsible for all healthcare and social care for their population.

In this study, we take the point of view of a commissioner that is responsible for all healthcare services for their population. From this point of view, what is missing in the VBHC theory is a way to utilize patient-level outcomes to steer value in a healthcare system that consists of multiple service providers with varying service portfolios and different degrees of specialization while the patient population is heterogeneous.

### The Use of Policy Instruments in Steering the Providers

The essence of steering is usually seen as power, control, and regulation. It is management across organizational boundaries, originally referring to means by which the state implements policies and transfers resources into desired actions, e.g., public services (15, 16). These means are divided into policy instruments that commonly include elements of regulating through legislation, economic means, and information (15–18).

In the context of this paper, steering refers to policy instruments that the commissioner uses to ensure that the outcomes of the public service create as much value as possible for the patients and the customers. Our main focus is on steering between commissioner and service provider, but we also touch on steering between commissioner and population.

Regulatory instruments define norms and acceptable behavior, and limit activities (19). Regulation is the most straight-forward and coercive policy instrument. The steered party (in this case the service provider) is obligated to do what the steering body (the commissioner) tells it to do. Depending on the national service structure, the commissioner can either regulate the provider using a contract between the two parties, or it can set the conditions the providers have to meet to qualify as service providers funded by the commissioner.

The economic means refer to the use of remuneration or deprivation of material resources. These means can either be understood narrowly (as incentives and disincentives) or widely (the allocation of resources in general) (16). In VBHC, the economic means are generally connected to incentive schemes for the service providers, where the service provider can basically decide whether the incentives or disincentives are big enough to be taken into account.

Information is a persuasive policy instrument, involving only the communication of claims and reasons instead of material resources or obligatory directives (16). In health care, information has a particularly important role, as the experts make independent decisions based on their best knowledge, and the availability of outcomes data extends this knowledge.

Dialogue is often considered a part of information as a policy instrument, since they are so closely connected in steering practices. In the context of this study, however, dialogue is seen as an independent tool, as we want to emphasize the need for two-way communication in steering. Dialogue builds trust between the parties and it also serves as a platform for co-production and co-creation, where the commissioner can gain knowledge about the provision of the services that it otherwise wouldn't gain (18).

When these policy instruments are applied in practice, they are always intertwined and used together. Regulation and economic means are often emphasized, especially when steering is seen as control, and the object of steering is seen as rational self-maximizer, calculating whether or not it should comply with the demands of the steering body (18). However, the object of steering has other motives than just deciding whether to comply or not: it may, for example, lack information that would make compliance more likely, or it may be distrustful toward the steering body, its policy goals, or the means used. In addition, the operations of the steered party may be too difficult or costly to monitor (20). Generally, the role of information and dialogue grow, as the steering structures become more complex, and the problems that ought to be solved become more wicked (18, 21).

### Value-Based Healthcare and Steering

When it comes to value-based steering of a healthcare system, the big question is how to combine steering with value (22, 23). In earlier literature, value-based steering and value-based management have held a somewhat different meaning (24, 25): value has been defined as any common goal, depending on the industry or context. In our paper, value-based steering means steering toward the specific value of health care systems defined by Porter—that is, cost-effectiveness.

Literature on VBHC and steering has focused largely on economic means of steering. Cattel et al. (26) design a theoretically preferred way of paying for value, focusing on the base payment. Roberts et al. (27) found that value-based payment may skew the system toward exacerbating health care disparities. Burns and Pauly (28) find that the adoption of value-based payment models has failed to reach its potential in improving outcomes. Chernew et al. (29, 30) and Choudhry et al. (31) focus on steering the patient by means of a value-based insurance, whereby the patient's co-payment is smaller for treatments where the cost-effectiveness is expected to be high. They postulate that such a setting does indeed steer the patient toward higher use of cost-effective therapies, yet evidence of health outcomes is still lacking. The ways that other means of steering could be coupled with outcomes data have not been extensively studied, even though there is an extensive body of research on e.g., information steering in healthcare. For example, Provan et al. (32) and Ferlie et al. (33) describe inter-organizational networks as a means of spreading information. Coiera et al. (34) and Kim et al. (35), on the other hand, describe suboptimal IT systems as a hindrance to reaching the full potential of a healthcare system from the point of view of patient outcomes.

The core of VBHC is collecting outcomes data and using it in a way that maximizes health benefits. Since the entire system is based on outcomes data, information has a two-fold role in steering. Firstly, it is the way for the commissioner to communicate its claims and reasons for the providers to achieve its overall goals; secondly, it is the most significant source of information behind the steering (36).

According to Porter's VBHC, outcomes and costs should be measured over patient episodes, making value a concept relevant on a patient level (3)—or patient segment level. Patient segmentation refers to grouping of healthcare users into smaller, more homogeneous subgroups (37), which is integral when designing patient-centered services. The commissioner/payer aims at maximizing value for all the patient segments, which means that its goals are defined through the goals relevant for each patient segment. The challenge for the commissioner is that in order to achieve its goals, it needs to operate through the providers, who provide the services for the patients and are in direct contact with them. However, in healthcare, the organization of service provision is largely based on specialization (general practitioners vs. tertiary care) and mode of operation (e.g., emergency care vs. elective care), which means that many producers participate in patient episodes, and each provider serves multiple patient segments. The solution suggested by Porter and Teisberg (1) is to organize production around patient segments. However, there are several reasons why this may not be feasible. In many countries, populations are simply too small to accommodate such specialized units, except for some specific elective procedures such as joint replacement surgeries. Furthermore, Enthoven et al. (38) argues that such a structure exacerbates the problem of silos, whereby patients with multiple morbidities or diffuse symptoms are left out. It is therefore necessary to fit together the goals of the patient segments, and the fragmented service provision.

How this fitting is to be done depends greatly on the context, in terms of hierarchical vs. market-driven model. If patients have extensive freedom of choice and money follows the patients (market-driven model), the commissioner needs to steer the patients, whereas in a hierarchical model the commissioning body needs to steer the producers. In practice the situation is often a combination of these two extremes: The commissioner has some control over producers but also the patient may have a possibility to choose between (a limited selection of) producers. In this paper, we focus on the means the commissioner has of steering the service providers.

The key question for the commissioning body to solve is the dilemma between the patient segment objectives (outcomes) and multiple producers participating in the production of services leading toward the outcomes—the same producers serving multiple patient segments. How to steer the service system/network toward the patient segment targets, when all steering mechanisms available are targeted toward individual producers? How to align the objectives of the producers with the objectives of the patient segments? The solution is simple when one producer is responsible for all the services for the patient segment. This is the case with many elective treatments and minor acute problems. However, with more complex problems, and often with chronic problems, the needs of the patients require participation from different professionals, often residing in different organizations.

### Objectives

The objective of this article is to answer the question: How can a commissioning body steer health services based on value in an environment, where the commissioner is responsible for the health services of the population with varying health service needs? The research question stems from a practical need: as Finland is about to undertake a major healthcare reform, a model for value-based steering is needed. Thus, the Prime Minister's Office of Finland commissioned this study.

To answer the question, we build a model for value-based steering for healthcare services. We approach the steering model by combining the principles of VBHC described above with policy instruments. Then, we move on to describing what steps need to be taken when implementing value-based steering. Finally, we provide a tool to help with the planning and implementing of value-based steering.

This sort of system-level value-based steering model has not been introduced before. Thus, this study contributes to both the academic discussion and the practice of healthcare commissioning. The model helps the commissioner utilize patient level data to steer the service providers, and thus implement value-based health care on a system level. Hence, it gives practitioners tools to understand and implement VBHC for the entire population, not only for a specific patient segment or a certain subset of producers. Also, the model considers broadly all different mechanisms that can be used for steering service providers, not only payment schemes.

## Methods

The model was developed between February 2019 and December 2019, as a part of a project funded by the Prime Minister's Office of Finland.

The study relies on Design Science. Introduced by Herbert Simon in 1969 (39), Design Science is by definition “a science of the artificial”—artificial meaning made by human as opposed to nature (39). Later, van Aken and Romme (40) defined the mission of Design Science as “to develop knowledge to support the solving of improvement of construction problems in a quest for improving the human condition.” Design Science is, in short, more solution-oriented and directly tied to practice than are natural sciences and humanities (40). According to Baskerville (41), “design science is directed toward understanding and improving the search among potential components in order to construct an artifact that is intended to solve a problem.” The artifact we seek to construct in this study is a model of value-based steering. We have chosen Design Science because of the nature of the problem at hand: we set out to construct a model, an artifact to fulfill a practical need, and Design Science is a discipline for doing precisely that.

**Figure 1:**
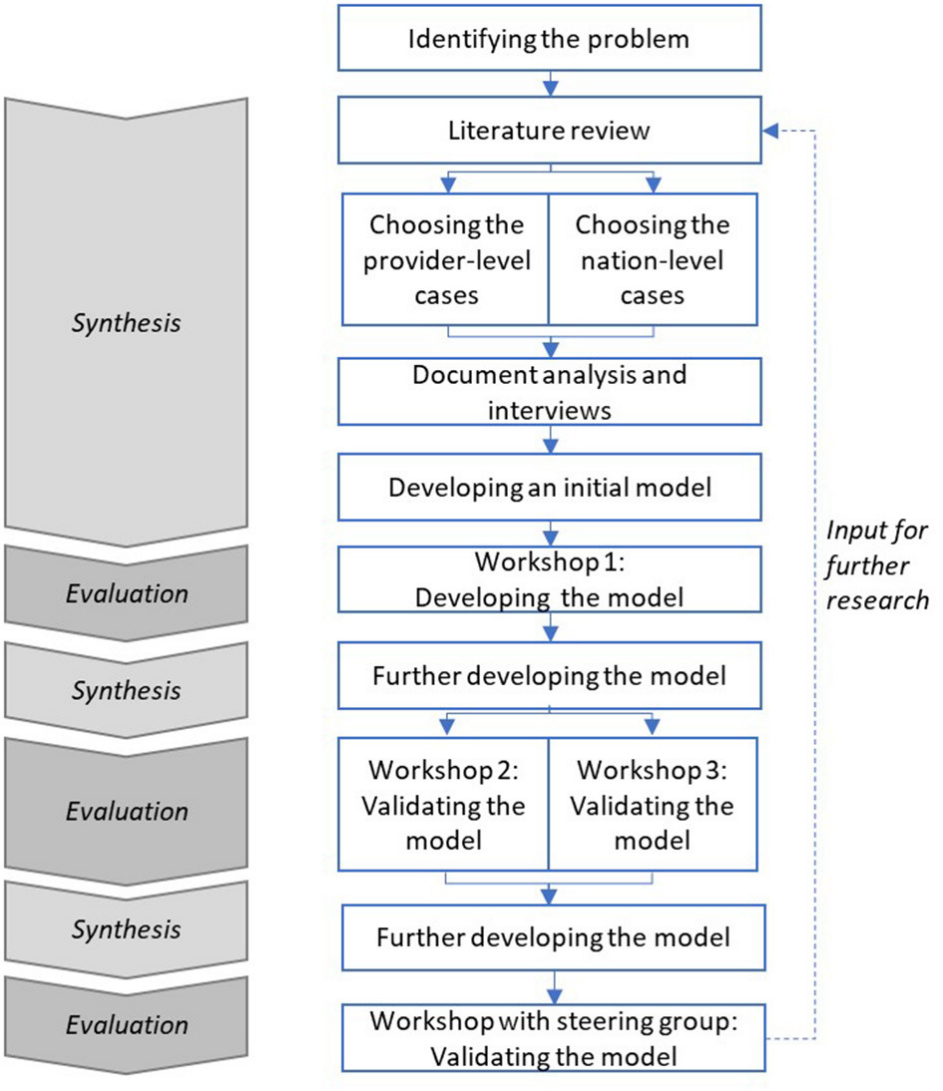
The steps of this study.

Taking a Design Science approach often involves a review of existing literature, followed by repeating cycles of synthesis and evaluation (40). Figure 1 highlights this cyclical nature of the process, describing the steps taken in this study.

In developing the model, we have utilized the principles outlined in the literature above. To build the basis of our model, we looked for examples of value-based steering in Finland, and to verify our findings we analyzed a few choice case examples of nation-level healthcare systems. Thus, we studied two kinds of case examples: (1) provider-level case examples, where a commissioner steers one (or a few) providers and (2) nation-level case examples, where the entire national healthcare system is examined. The provider-level case examples were chosen primarily from Finland, and secondarily from other countries, as the steering model is aimed for use in Finland primarily. The Finnish case examples were chosen based on the researchers' extensive knowledge of such examples in Finland, with the aim of covering different services and patient segments as well as possible. While our selection of Finnish cases is not exhaustive, it does represent the current situation in Finland. The cases we chose not to analyze here are very similar to the ones included. They represent either public-private partnerships or the private sector, because no examples of systematic value-based steering were available within the public sector. They are complemented by one foreign example (Santeon Hospitals), because there are no current case examples of value-based steering in secondary care in Finland. All of these cases were researched *via* document analysis, and semistructured interviews (Supplementary Material A) were undertaken to complete the analysis framework (Supplementary Material B) where necessary.

To verify our findings and see whether similar principles apply also on national level, we referred to Papanicolas et al. (42) for a nation-level comparison of life expectancy and healthcare expenditure. Furthermore, we chose additional cases based on our own knowledge and an interview with prof. Paul Lillrank (The Institute of Healthcare Engineering, Management and Architecture, Aalto University). We followed the same process for these cases as the provider level cases, described in the preceding paragraph. Our reasoning for choosing each case is detailed in Supplementary Material C.

Based on the literature and the case examples, we created an initial model for value-based steering. The model was further developed in two focus group workshops with two commissioning bodies (two Finnish regional healthcare authorities: Pirkanmaa and Central Finland) and one workshop with attendees from all around Finland. The job titles and organizations of the attendees of each workshop are listed in Supplementary Material D. This work was regularly validated by a steering group that consisted of public officers from different Finnish ministries: Ministry of Social Affairs and Health, Ministry of Finance, and Ministry of Economic Affairs and Employment.

## Results

In Table 1, we have described how different means of steering are used in each provider-level case example. These case examples are very consistent in highlighting what works in terms of policy instruments: the economic means that are emphasized as the main policy instrument in VBHC literature are utilized in each one, but their steering capacity is very limited—financial incentives based on outcomes only cover between 1 and 5% of the remuneration. Neither do the case examples rely on regulation: regulation is only used to mandate the measuring of outcomes. Instead, the cases are all based on continuously utilizing the information: on regularly evaluating the outcomes, and on systematic dialogue about the outcomes to ensure the continuity of the steering process, and, in the end, the cost-effectiveness of the services. Thus, we conclude that information and dialogue are central in value-based steering practices.

**Table 1:** Findings from the provider-level case examples.

**Case**	**Economic means of steering**	**Regulation**	**Information and dialogue**
“Tesoma” primary healthcare, dental care and social services outsourcing; Tampere, Finland	1.6% of compensation tied to outcome metrics	Outcome measurement is obligated in the outsourcing agreement.	Information and dialogue are utilized by sharing and discussing the outcomes data regularly.
Korpilahti and Tikkakoski (and Säynätsalo) primary health care outsourcing; Jyväskylä, Finland	1 and 3.5% (for Korpilahti and Tikkakoski, respectively) of compensation tied to outcome metrics	Outcome measurement is obligated in the outsourcing agreement.	Information and dialogue are utilized by sharing and discussing the outcomes data regularly.
“Kotitori” elderly care outsourcing with integrator; Tampere, Finland	2% of compensation tied to outcome metrics	Outcome measurement is obligated in the outsourcing agreement.	Information and dialogue are utilized by sharing and discussing the outcomes data regularly.
Pohjola Hospital and Pohjola Insurance; Finland	None	Pohjola Insurance, as the owner of Pohjola Hospital, obligates measuring of outcomes.	Information and dialogue are utilized by sharing and discussing the outcomes data regularly.
Santeon hospitals; the Netherlands	5% of compensation tied to outcome metrics	Outcome measurement is obligated in the agreement.	Information and dialogue are utilized by sharing and discussing the outcomes data regularly.

To verify these findings and see if similar principles also apply on nation-level, we analyzed a three nation-level cases: Japan, Singapore, and NHS England.

We expected to find value-based steering practices behind the good results. However, we found that there was little evidence of any systematic steering behind the good outcomes. In Japan and Singapore, the outcomes seem to stem from nutritional habits, favorable genetics etc. instead of systematic national steering. In the case of NHS England, we found that there was systematic steering based on cost-effectiveness data, and even though monetary incentives were an important part of the model, resource steering is far from the only steering mechanism employed. Thus, our takeaway from the nation-level case examples was that financial means of steering are useful yet not sufficient on their own. This was consistent with our findings in the provider-level case examples.

Thus, we based our steering model on these findings: economic means are important but not sufficient on their own. Regulation can be used to obligate measuring outcomes, and this outcomes information is then shared and discussed.

### Value-Based Steering Model

We introduce a value-based steering model, which illustrates how the commissioner can steer value through the different policy instruments: regulation, economic means, information, and dialogue. It should be noted that while this paper focuses solely on value-based steering, in reality the commissioner may have other goals apart from value, such as equity, access, and safety, and thus value-based steering is applied simultaneously with other objectives of steering.

Based on the case analysis we identified three essential parts to the model: (1) the principles of value-based steering; (2) the steering process; and (3) the Value-Steering Canvas.

**Figure 2:**
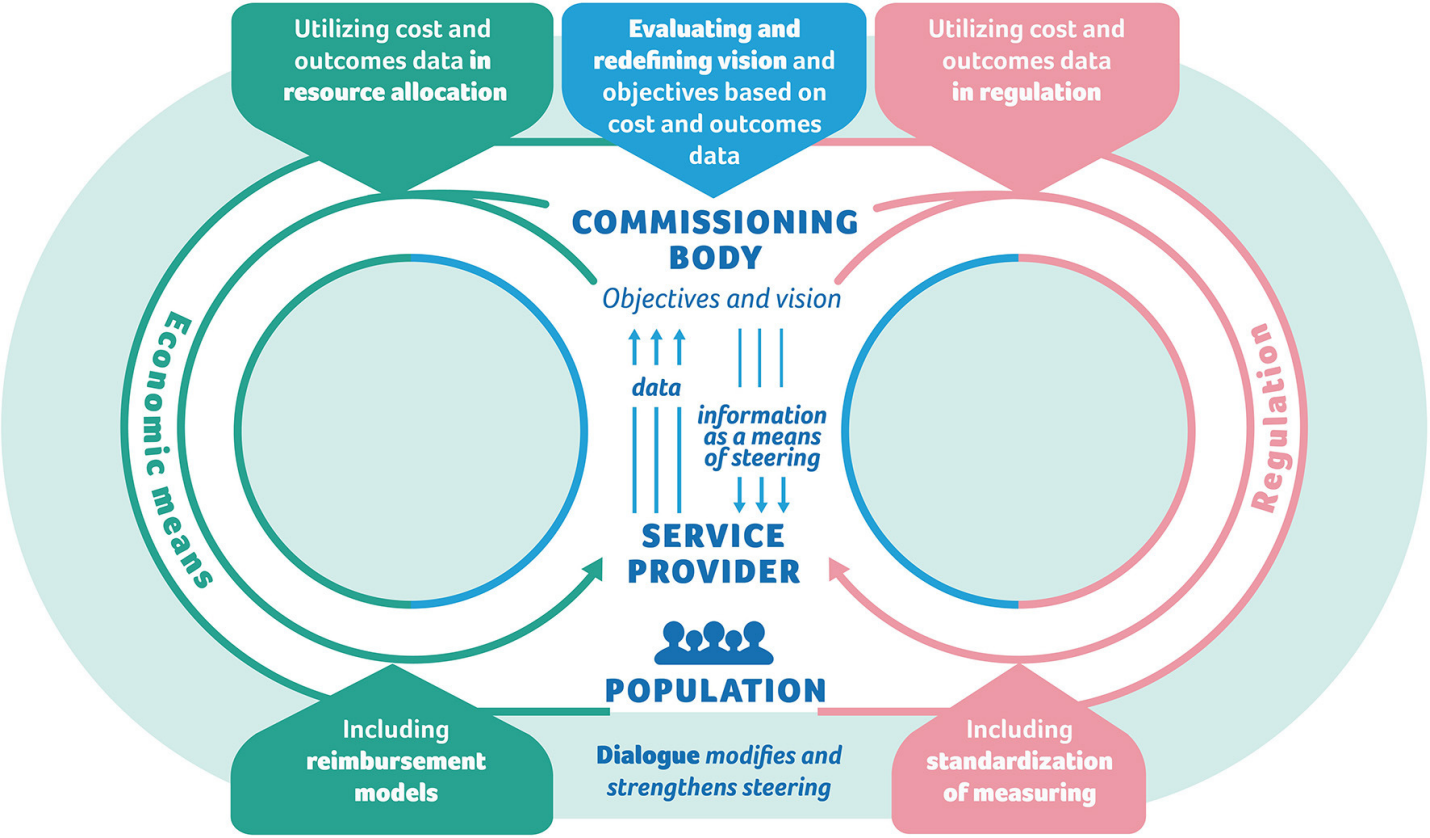
The principles of steering.

Figure 2 illustrates the principles of steering. Based on the cases, outcomes and cost data collected on a patient-level is the engine of the entire steering system, and all the policy instruments are connected to it. The commissioner obliges the service providers to collect the data, which are then pooled. The commissioner utilizes the data to inform the service providers and professionals about the outcomes of the services, and to allocate resources. The commissioning body can create reimbursement models for service providers based on their outcomes or use it to reframe the budget, which can mean either rewarding for performance or supporting services where outcomes are below target. Dialogue builds trust and mutual understanding, and modifies and strengthens steering and fills the inevitable gaps in the data.

**Figure 3:**
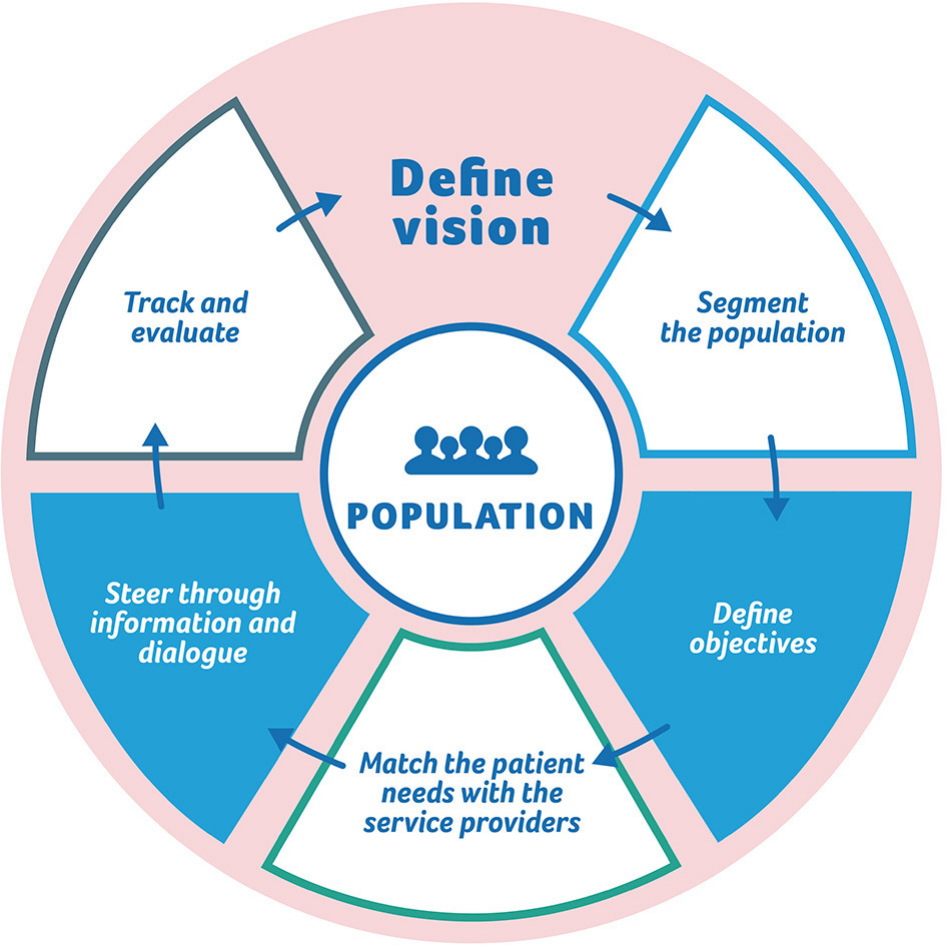
The process of steering.

Figure 3 illustrates the process of steering. It describes how the steering practices are built and maintained. First, the commissioner defines concrete large-scale objectives. Then the population is segmented (based on their service needs) and objectives are defined for each segment and for each type of service provider.

Based on the case analysis, the main difficulties in creating a value-based service system is aligning objectives of patient segments with objectives of service providers: the system is based on patient segmentation but the steering is directed at the service providers. A service provider may serve various patient segments with varying objectives. As an example, a health center may be responsible for non-severe acute patients and chronic patients.

In addition, a single service provider may take care of only part of the care or service pathway. Thus, service pathways will require integration and coordination. In practice, this means that the role of the service provider in the care pathway has to be taken into account when defining the outcomes objectives. Also, the network of service providers is developed through interaction between the commissioner and the service providers—that is, using information and dialogue as policy instruments. Finally, the content and the process of steering, as well as the objectives, are evaluated and renewed based on the outcomes data.

The process of steering answers the question of how individual-level outcomes and service providers caring for many different patient segments can be coupled together.

**Figure 4:**
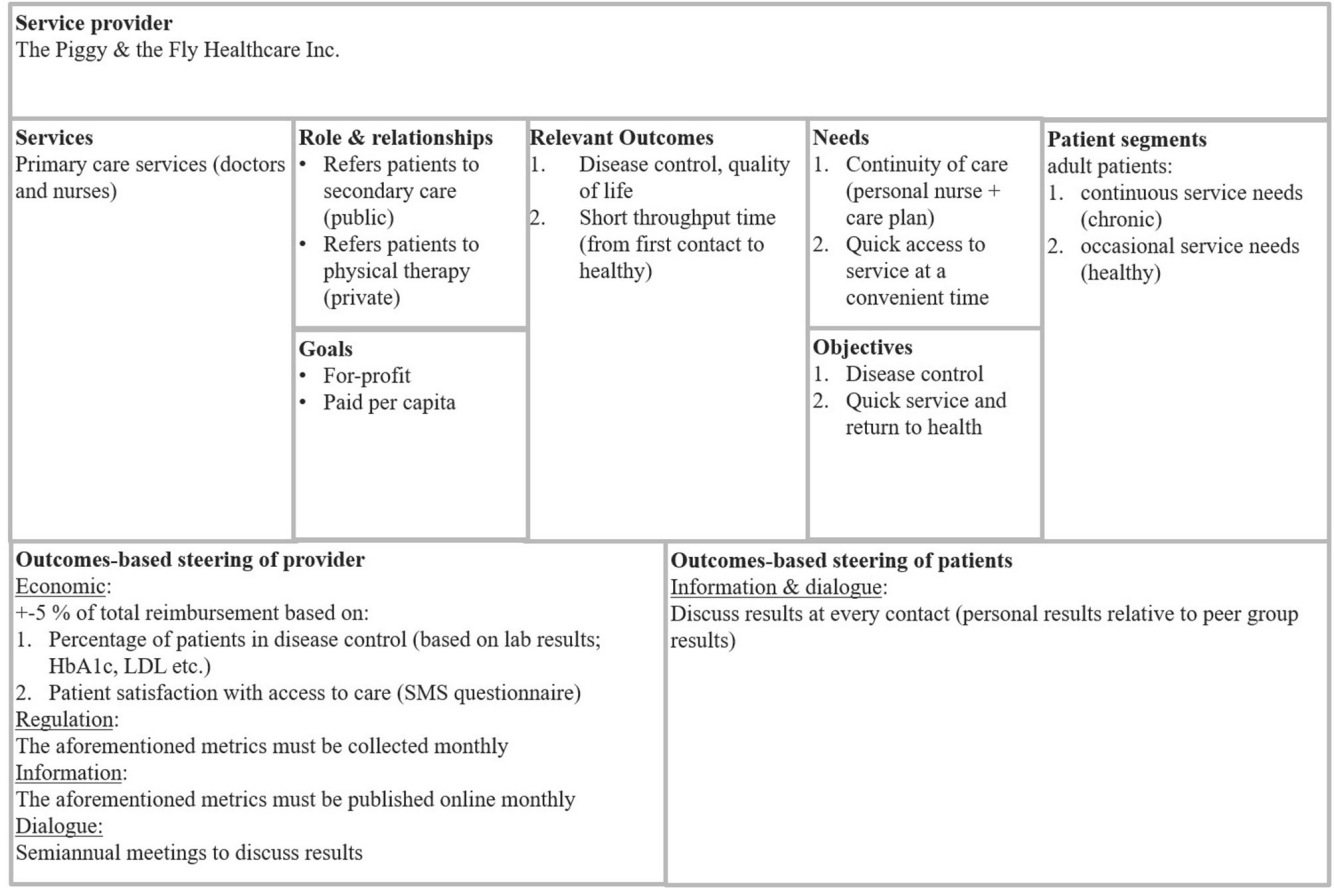
The value steering canvas.

Figure 4 illustrates the third part of the steering model: the Value Steering Canvas (VSC), which can be used as a tool when designing and implementing value-based steering practices. We have modeled the VSC loosely after the Business Model Canvas, originally introduced in 2005 by Osterwalder (43). The VSC is a tool for the commissioner to design the details of the value-based steering of both the provider and the patients, and it provides a tool to help the alignment of the goals of the patient segments with the incentives of the providers.

The VSC combines the most relevant attributes of the service provider and the patients. It takes into account the goals of the service provider as well as the needs of the patient segments. It includes 9 elements, or boxes. The titles of the boxes as well as the descriptions of their intended content are described next. It should be noted that the detailed content of the boxes in Figure 4 is only meant as an example of the level of description we suggest the commissioners and service providers use for this tool.

On the top of the VSC, the provider is named. The left-hand side of the canvas represents the service provider. “Services” describes the services produced by the service provider for the commissioner. Only services potentially aimed at the same patient segments should be listed here—in case the provider is large, there may be many more services produced, but unless the patient segments overlap, these should be ignored (or rather, they are the building blocks of another VSC). When the commissioner and the object of steering are different organizations, there is usually a contract between them, regulating the service production. As a rule of thumb, there should be one VSC per each contract. For example, the services listed here could be outsourced primary healthcare outpatient services. While the provider may also produce home care, unless this is a part of the same contract or otherwise tightly coupled to the provision of primary healthcare services, this should be ignored. “Role and relationships” describes the role of this particular service provider within the network of service providers serving the same patients, and its relationships to the aforementioned. For example, other service providers in the care path of the same patients should be mentioned here. In our example of primary care, this box could include secondary care providers to whom this provider refers patients. “Goals” describes the ultimate goals of the service provider. These depend on whether the provider is for-profit or non-profit, and whether it is part of the same organization as the commissioner (internal) or not (external). These attributes affect the intrinsic incentives of the provider.

The right-hand side, then, represents the patients. “Patient segments” describe the patient segments served. Again, only the relevant ones should be included. In our example, the provider serves only adult patients, which should be mentioned in this box. The patients should be segmented in a meaningful way, preferably (as outcomes are of relevance here) based on their service needs: in a way that is meaningful in terms of their expected outcomes. “Objectives” describe the relevant objectives for the patient segments. The objectives should be thought of in terms of outcomes—they should be something that conceivably leads to better outcomes. For example, for the chronically ill, good control of the disease is usually a meaningful objective, as it is in many cases statistically linked to fewer adverse outcomes—for example, good HbA1C control in diabetes mellitus is linked with fewer vascular and renal complications. It should be noted that the objectives usually are different for each patient segment. “Needs” describe the high-level needs of the patient segments, that is, what they need in order to reach the objectives. Again, these are usually different for each segment. For example, for the diabetes patients to reach good control, they need continuity of care which could mean their own personal nurse (and/or physician) and a written care plan.

“Relevant outcomes” are a synthesis of the different sides: it is the overlap of what the patients need and what the service provider does. The outcomes are at the heart of the steering model: they are what is measured, and they are what the incentives are planned to aim at.

“Outcomes-based steering of the provider” includes the outcomes metrics and the steering instruments through which the commissioner steers the service provider. This includes all the steering instruments: not only economical instruments (bonus/sanction model), but also information (measuring outcomes and sharing the information), dialogue (forums for discussing the outcomes), and regulation (contracts and other norms). The economical instruments often revolve around a bonus/sanction model, where economic incentives are coupled with outcome measures. Such is the case for example for Santeon Hospitals, which receives 95–105% of base tariff from the payer based on the outcomes reached.

The coercive policy instruments are often thought to be the strongest ones, but regulation has its limitations. Each contractual obligation needs a potential sanction for failure to comply, otherwise it is meaningless. As such, it is essentially a bonus/sanction model without the bonus (16). Even so, things that can and should be regulated from the point of view of value-based steering, include measuring the outcomes and sharing the data: only comprehensive data can fuel the steering engine, ensuring that the policy instruments relying on the outcomes data operate on reliable information.

According to our interviews regarding the Finnish case examples, healthcare professionals usually have a high moral standard and an obligation to their patients, which is a strong motivator. Therefore, information steering in the form of sharing data may be a particularly efficient policy instrument in the context of health care. A simple thing such as measuring disease control and reporting it per professional is usually enough to make the professionals benchmark against each other, striving to learn from their more successful counterparts, according to the interviews. Any manager reporting such results to their employees should handle it with great discretion, so as to avoid ranking the professionals or blaming the ones with less good outcomes. Case-mix should be taken into consideration, and whenever possible, the set of measures should be so all-encompassing that everyone excels at something.

“Outcomes-based steering of the patients” describes the means of steering the patient, and its practicalities and responsibilities. Both commissioner and service provider may do their part in steering the patient toward better outcomes. Typical things to consider here are lifestyle choices, such as nutrition and exercise, which are paramount for achieving good outcomes, yet both the commissioner and the service provider have a limited possibility of influencing them, at best.

We suggest the VSC be used by the commissioner for creating value-based steering practices for a service provider. The VSC can be used as a blueprint for negotiations between commissioner and service provider, thus creating a win-win situation.

## Discussion

This study proposes a steering model that is suitable for value-based health care. It answers the question: How can a commissioning body steer value-based health services in an environment where the commissioner is responsible for all health services of a certain population with various needs? The study is based on Finland where healthcare is a public service and the system is mainly tax-funded, and currently the commissioner and the provider are usually the same organization. The steering model can be used in any setting where there is a commissioning body (government, insurance company, etc.) and service providers (public or private entities). It is even more easily applicable in countries where these are separate organizations, as resource steering is likely more applicable between organizations than within them.

Earlier studies have focused more on the reimbursement based on outcomes (22) and paid less attention to other steering mechanisms. In steering such a complex system, changes are required in all steering mechanisms as well as multiple levels from basic principles of steering to specific tools to steer a single service provider. We described the main principles, the process, and a tool for value-based steering in this article, taking into account all policy instruments, also non-economic. Detailed ideas on the practicalities of implementing and utilizing non-economic policy instruments have been recently described by e.g., WHO (44) and EIT (45).

We mentioned building and maintaining provider networks as a part of the steering process. We believe value for patients could be a common goal around which such networks could be built, and this could potentially amplify the steering efforts of the commissioner, through e.g., sharing information. Earlier literature (32, 33) describes how a commissioner can facilitate the evolution of such networks. As for IT systems, we suggest they be taken into consideration, as their optimal performance seems to be a prerequisite for reaching optimal patient outcomes (34, 35).

The original value-based approach (1) needs to be widened from specific health problems to taking also e.g., prevention and multimorbidity into account when building a steering system for the whole population. On a system level, the data must also enable comparison of outcomes and costs between different patient segments, in addition to measuring relevant outcomes for each patient segment. Value-based steering objectives should be aligned with objectives of public health.

A crucial limitation to implementing value-based healthcare is the lack of individual-level outcomes data. However, our model includes obligating the service providers to gather such data where it is not yet available. As outcomes data is at the heart of the model, obligating measuring outcomes should be the first step. Furthermore, we suggest further research on how process metrics can be used as a proxy for outcomes—so as to circumvent the problem of lack of data.

In practice, we suggest that some of Finland's 21 regions start by obligating outcomes measurement in a chosen patient segment, then proceed to implement the model for that segment, and gradually include more patient segments as the data and the knowhow accrue. Similarly, the steering mechanisms can be developed incrementally, starting with information and dialogue, and only later developing the rules and incentives based on the experiences of utilizing value-based information.

Another obstacle is the complexity of the patients and the providers, especially as healthcare systems shift toward integrated care instead of focusing on a single diagnosis. However, the present study is based on Finland where care is already integrated, especially in primary health care, so we have taken this into account when designing the model. We recognize that our model is a simplification of reality, as models always are (46). Even so, our model describes the elements needed for value-based steering.

We expected to find useful value-based steering practices from literature, but found very little. In the case of Japan, we used high life expectancy as an outcome, because it is the most widely available outcomes data. However, it seems that life expectancy is too vague a metric to be used as an indicator of healthcare system performance, because it is influenced by many factors. Murray (47) cites “favorable cultural heritage of dietary risk factors and physical activity” among reasons for Japan's high life expectancy. Therefore, we suggest using more specific outcome metrics in the future when striving to recognize well-functioning healthcare systems. Furthermore, our analysis focused on only three case examples and was by no means an exhaustive literature review. We suggest a more thorough review of nation-level cases of value-based steering for future research.

## Conclusions

We constructed a conceptual model for value-based steering by a commissioner. Further studies are required to test the model. In Finland, the commissioning bodies can start using value-based steering without changes in legislation or in the present service system.

Also, a key requirement for value-based steering is patient-level information of outcomes and costs—all value-based steering mechanisms are based on this information. In Finland as well as internationally, the missing piece is the outcomes data—especially PROMs are still rarely collected. In addition to commissioners obligating data collection as a part of the model, governments could take an active role in setting goals for PROM collection, as has been done e.g., in the Netherlands (48). Another challenging issue is the co-creation of value: patients contribute to value creation also outside of the healthcare system. The requirement for extensive knowledge, linking health outcomes to the full cycle of treatment and its costs, significantly challenges the adoption of the value-based steering system (23).

If we overcome these hurdles, we can improve our healthcare systems by making use of outcomes information while recognizing the shortcomings of Porter's theory.

## Data Availability Statement

The original contributions presented in the study are included in the article/Supplementary Material, further inquiries can be directed to the corresponding author/s.

## Author Contributions

LP, PT, R-LL, and HT conceived the article, contributed to analysis, workshops, production of the manuscript, and approved submission. All authors contributed to the article and approved the submitted version.

## Funding

This study was funded by Prime Minister's Office of Finland.

## Conflict of Interest

The authors declare that the research was conducted in the absence of any commercial or financial relationships that could be construed as a potential conflict of interest.

## Publisher's Note

All claims expressed in this article are solely those of the authors and do not necessarily represent those of their affiliated organizations, or those of the publisher, the editors and the reviewers. Any product that may be evaluated in this article, or claim that may be made by its manufacturer, is not guaranteed or endorsed by the publisher.
