# Supplementary material for: A Value-Based Steering Model for Healthcare
Source: Front Health Serv. 2021 Nov 26;1:709271. doi: 10.3389/frhs.2021.709271 (PMC10012620; doi:10.3389/frhs.2021.709271)
Supplement: Supplementary file 1 [file Table_1.DOCX]

Supplementary Material A: Interviewees and documents analysed

| Level | Case | Examples of documents analysed | Interviewees |
| --- | --- | --- | --- |
| Provider-level | Tesoma, Tampere, Finland | Procurement documents, available at request from the City of Tampere | Service Director, Outpatient Services, City of Tampere;  CEO, Coxa Hospital for Joint Replacement |
|  | Korpilahti and Tikkakoski, Jyväskylä, Finland | Procurement documents, available at request from the City of Jyväskylä | Service Director, Outpatient Services, City of Jyväskylä |
|  | Kotitori, Tampere, Finland | Procurement documents, available at request from the City of Tampere | Service Director, Outpatient Services, City of Tampere;  Senior Advisor, Nordic Healthcare Group |
|  | Pohjola Hospital, Finland | G3 – Vaikuttavuuden johtaminen sosiaali- ja terveydenhuollossa. Aalto University, 2018. Available at <https://www.aalto.fi/sites/g/files/flghsv161/files/2019-04/G3-julkaisu.pdf> | CEO, Pohjola Hospital |
|  | Santeon, the Netherlands | Okunade et al. 2017. Collaborating for value: the Santeon Hospitals in the Netherlands. Available at  <http://www.ichom.org/wp-content/uploads/2013/10/Santeon_Case_Study_Final.pdf> | Director, Santeon Hospitals, the Netherlands |
| Nation-level | Singapore | E. Mossialos, A. Djordjevic, R. Osborn, and D. Sarnak (eds.), International Profiles of Health Care Systems, The Commonwealth Fund, May 2017. | Associate Professor, National University of Singapore, School of Public Health |
|  | Japan | E. Mossialos, A. Djordjevic, R. Osborn, and D. Sarnak (eds.), International Profiles of Health Care Systems, The Commonwealth Fund, May 2017. | Professor, Department of Industrial Engineering and Management, Aalto University, Finland |
|  | NHS England | NHS England. (2018). Report of the Review of the Quality and Outcomes Framework in  England. Available at <https://www.england.nhs.uk/wp-content/uploads/2018/07/quality-outcome-framework-report-of-the-review.pdf>  Raleigh VS. (2012). Pick ‘n’ mix: an introduction to choosing and using indicators. The King’s Fund. Available at <https://www.slideshare.net/kingsfund/outcomes-ccg-slide-pack-june-2012finalforslideshare> | Healthcare Consultant, Nordic Healthcare Group / M.Sc. (Health Economics), London school of Economics Alumn |
