# Supplementary material for: A Value-Based Steering Model for Healthcare
Source: Front Health Serv. 2021 Nov 26;1:709271. doi: 10.3389/frhs.2021.709271 (PMC10012620; doi:10.3389/frhs.2021.709271)
Supplement: Supplementary file 2 [file Table_2.DOCX]

Supplementary Material B: Analysis framework for the cases

| Who steers whom and towards what objectives? | What is the organization that is doing the steering? |
| --- | --- |
|  | What is the organization that is being steered (object of steering)? |
|  | What are the objectives of the organization that is doing the steering? |
|  | What sort of social and/or healthcare services are being provided? |
|  | Who are the end customers? |
| Outcomes measurement | Which metrics are used? |
|  | Is measuring systematic over time? |
|  | How comprehensive is the measuring? (what percentage of patients) |
|  | Who does the measuring and how, in practice? |
| How is outcomes data utilized with… | …regulatory instruments of steering? |
|  | …economic means of steering? |
|  | …information steering? |
|  | …dialogue as a means of steering? |
| How do these steering practices work? | What are the positives? |
|  | What are the negatives? |
|  | What results have been attained? |
