# Supplementary material for: A Value-Based Steering Model for Healthcare
Source: Front Health Serv. 2021 Nov 26;1:709271. doi: 10.3389/frhs.2021.709271 (PMC10012620; doi:10.3389/frhs.2021.709271)
Supplement: Supplementary file 3 [file Table_3.DOCX]

Supplementary Material C: The reasoning for choosing each case example

| Level | Case | Reasoning for choosing the case example |
| --- | --- | --- |
| Provider-level | Tesoma, Tampere, Finland | The only known example of alliance model used to procure services in Finland; also partially outcomes-based compensation for the provider. |
|  | Korpilahti and Tikkakoski, Jyväskylä, Finland | One of the few examples of outcomes-based healthcare service procurement in Finland. |
|  | Kotitori, Tampere, Finland | One of the few examples of outcomes-based elderly care service procurement in Finland. |
|  | Pohjola, Finland | Pohjola Hospital is the only Finnish hospital owned by an insurance company, and it utilizes value-based steering (Peltokorpi et al. 2018. How can the integration of a payer and a provider create value in health care? Published Online:9 Jul 2018 https://doi.org/10.5465/AMBPP.2018.14075abstract) |
|  | Santeon, the Netherlands | An example of outcomes-based compensation in specialized health care. |
| Nation-level | Singapore | Singapore is known to have a unique healthcare system, and their outcomes are on par with many developed countries while spending is considerably smaller (https://www.moh.gov.sg/resources-statistics/educational-resources/achieving-more-with-less---singapore's-healthcare-expenditure#:~:text=Singapore%20is%20achieving%20similar%20outcomes,comparable%20with%20many%20developed%20countries) |
|  | Japan | In Papanicolas’ report (36), Japan has the highest life expectancy at birth and the third lowest healthcare spending per capita, among the 11 high-income countries studied. |
|  | NHS England | The NHS is a well-known European example of outcomes-based steering. |
