# Supplementary material for: A Value-Based Steering Model for Healthcare
Source: Front Health Serv. 2021 Nov 26;1:709271. doi: 10.3389/frhs.2021.709271 (PMC10012620; doi:10.3389/frhs.2021.709271)
Supplement: Supplementary file 4 [file Table_4.DOCX]

Supplementary Material D: Workshop attendees

Workshop 1, June 12th 2019, Helsinki, Finland

Senior Adviser, Industrial Affairs, Ministry of Economic Affairs and Employment of Finland

Manager of Development and Communications, Municipality of Kirkkonummi

Leader of Change, Health and Social Services Reform, Uusimaa Regional Council

Ministerial Adviser, Ministry of Economic Affairs and Employment of Finland

CEO, Council of Oulu Region

Director, Employment and Economic Development Office of Pirkanmaa

Head of Adult Social Services, Päijät-Häme Health and Social Care District

Chief of Unit, Centre for Economic Development, Transport and the Environment of Lapland

Quality Manager, Finnish Food Authority

Senior Social Worker, Family and Social Services, City of Turku

Medical Adviser, Association of Finnish Local and Regional Authorities

Development Manager, City of Tampere

Chief Architect, SoteDigi Oy

Senior Specialist, Ministry of Finance

Employment Coordinator, City of Parkano

Controller, City of Joensuu

Head of employment Services, City of Forssa

Project Manager, City of Forssa

Service Manager, City Jyväskylä

Coordinator, Employment and Growth Services, City of Tampere

Chief Development Physician, Central Finland Health Care District

Strategy Director, Development and Administration centre for ELY Centres and TE Offices

Planning Manager, City of Tampere

Director of Uusix Workshops, City of Helsinki

Project Manager, The Council of Pirkanmaa Region

Chief Medical officer of Clinical Auditing, Tampere University Hospital

Programme Manager, Regional Council of Central Finland

Director, Centre for Economic Development, Transport and the Environment of Lapland

Service Manager, City of Jyväskylä

Director, Employment Services, Municipality of Kirkkonummi

Leading IT Architect, City of Helsinki

Manager, Employment Services, City of Kouvola

Manager, Immigration and Employment Services, City of Tuusula

Senior Adviser, Centre for Economic Development, Transport and the Environment of Northern Karelia

Manager, Employment Services, City of Kangasala

Director, Employment and Economic Development Centre of Central Finland

Project Director, Provider Steering and Contracts, Uusimaa Regional Council

Director of Architecture, Vimana Oy

National Coordinator, Social and Health Care, Employment and Economic Development Centre of Finland Proper

Rehabilitation Coordinator, Municipality of Kirkkonummi

Workshop 2, October 8th 2019, Tampere, Finland

Service Director, City of Tampere

Development Manager, City of Tampere

Project Director, City of Tampere

Senior Social Worker, City of Tampere

Director of elderly Care Services, City of Tampere

Social Worker, City of Tampere

Project Manager, City of Tampere

Development Manager, City of Tampere

Procurement Officer, City of Tampere

Planning Manager, City of Tampere

Workshop 3, October 16th 2019, Jyväskylä, Finland

CEO, Central Finland Health Care District

Director of Nursing Excellence, Central Finland Health Care District

Director of Social and Healthcare Reform, Region of Central Finland

Director of Social and Healthcare Services, City of Jyväskylä

Financial Manager, City of Jyväskylä

Development Manager, Central Finland Health Care District

Chief Development Physician, Central Finland Health Care District
